# Development of a Multicomponent Microbiological Soil Inoculant and Its Performance in Sweet Potato Cultivation

**DOI:** 10.3390/microorganisms11040914

**Published:** 2023-03-31

**Authors:** Viktor Dávid Nagy, Anuar Zhumakayev, Mónika Vörös, Ádám Bordé, Adrienn Szarvas, Attila Szűcs, Sándor Kocsubé, Péter Jakab, Tamás Monostori, Biljana D. Škrbić, Edina Mohai, Lóránt Hatvani, Csaba Vágvölgyi, László Kredics

**Affiliations:** 1 Department of Microbiology, Faculty of Science and Informatics, University of Szeged, Közép fasor 52, 6726 Szeged, Hungary; 2 Faculty of Agriculture, University of Szeged, Andrássy Street 15, 6800 Hódmezővásárhely, Hungary; 3 Faculty of Technology, University of Novi Sad, Bulevar cara Lazara 1, 21000 Novi Sad, Serbia

**Keywords:** *Trichoderma*, *Pseudomonas*, *Bacillus*, *Arthrobacter*, microbiological soil inoculant, biocontrol, plant growth promotion, sweet potato

## Abstract

The cultivation and consumption of sweet potato (*Ipomoea batatas*) are increasing globally. As the usage of chemical fertilizers and pest control agents during its cultivation may lead to soil, water and air pollution, there is an emerging need for environment-friendly, biological solutions enabling increased amounts of healthy crop and efficient disease management. Microbiological agents for agricultural purposes gained increasing importance in the past few decades. Our goal was to develop an agricultural soil inoculant from multiple microorganisms and test its application potential in sweet potato cultivation. Two *Trichoderma* strains were selected: *Trichoderma ghanense* strain SZMC 25217 based on its extracellular enzyme activities for the biodegradation of plant residues, and *Trichoderma afroharzianum* strain SZMC 25231 for biocontrol purposes against fungal plant pathogens. The *Bacillus velezensis* strain SZMC 24986 proved to be the best growth inhibitor of most of the nine tested strains of fungal species known as plant pathogens, therefore it was also selected for biocontrol purposes against fungal plant pathogens. *Arthrobacter globiformis* strain SZMC 25081, showing the fastest growth on nitrogen-free medium, was selected as a component with possible nitrogen-fixing potential. A *Pseudomonas resinovorans* strain, SZMC 25872, was selected for its ability to produce indole-3-acetic acid, which is among the important traits of potential plant growth-promoting rhizobacteria (PGPR). A series of experiments were performed to test the selected strains for their tolerance to abiotic stress factors such as pH, temperature, water activity and fungicides, influencing the survivability in agricultural environments. The selected strains were used to treat sweet potato in two separate field experiments. Yield increase was observed for the plants treated with the selected microbial consortium (synthetic community) in comparison with the control group in both cases. Our results suggest that the developed microbial inoculant has the potential to be used in sweet potato plantations. To the best of our knowledge, this is the first report about the successful application of a fungal-bacterial consortium in sweet potato cultivation.

## 1. Introduction

The environment-friendly methods of crop management are more important than ever, especially since the European Commission has been planning to reduce the chemicals used in the agriculture to 50% by 2030 [1]. The use of chemical fertilizers and pest control agents in crop management has led to soil, water and air pollution [2]. These chemical compounds are harmful to the environment and human health [3,4,5]. Using microorganisms in agriculture instead of chemical agents could be the future of crop management. In the past few decades, beneficial microorganisms were widely used in agriculture for fertilization, plant growth promotion and biological control of bacterial or fungal phytopathogens and eventually nematodes or pests [6,7,8]. The development of multicomponent agricultural soil inoculants is a reasonable approach [9,10], as such inoculants may be able to cope with a wide range of agricultural needs.

Many biological soil inoculant products contain *Trichoderma* component(s) due to their beneficial effect on both the soil and the plants. These filamentous fungi take part in the degradation of plant residues in the soil and contribute to the maintenance of the soil nutrient level [11]. *Trichoderma* strains are also beneficial by promoting plant growth and inducing Systemic Acquired Resistance (SAR) in plants, furthermore, they are useful agents against plant pathogens—especially fungi—through their antagonistic abilities by the production of secondary metabolites, efficient competition for space and nutrients and eventual mycoparasitism [12].

The genera *Bacillus* and *Pseudomonas* are among the most popular bacterial components of soil inoculants. Competition with other microorganisms in the soil for nutrients and space, antibiosis by the production of secondary metabolites such as lipopeptides, the induction of Systemic Acquired Resistance (SAR) in plants and plant growth promotion via indole-3-acetic acid (IAA) production are among the beneficial effects of *Bacillus* species [13,14,15]. *Pseudomonas* species are also frequently used in the agriculture for plant growth promotion and regulation through IAA, siderophore production, and the facilitation of nutrient uptake by phosphate solubilization [16].

The cultivation of sweet potato (*Ipomoea batatas*) gained increasing popularity during the past few decades as it is a rich source of nutrients [17], especially β-carotene and anthocyanin [18], easy to cultivate due to its adaptability to a wide range of soils and climates [19], known for high yield, and its roots, shoots and leaves are also edible [20]. Beneficial microorganisms have already been tested for biocontrol purposes, for the improvement of plant tolerance to abiotic stress, as well as for growth promotion of sweet potato plants [21,22,23,24,25].

The aim of this study was to develop a multicomponent agricultural soil inoculant. To realize this aim, our objectives were to characterize fungal and bacterial isolates for various properties (ecophysiological features, biocontrol potential, nitrogen fixation, phosphorus mobilization, polysaccharide degradation, pesticide tolerance), to assemble a synthetic community from microbial strains selected based on their promising features, and to examine its performance in sweet potato cultivation.

## 2. Materials and Methods

### 2.1. Microorganisms

A total of seven *Trichoderma*, 13 *Bacillus*, 20 potentially N_2_-fixing and five glyphosate-tolerant bacterial strains deriving from various agricultural soils of the Hungary-Serbia cross-border region were included in this study [26,27,28] (Table 1). The isolation of *Trichoderma* strains was performed by suspending 1 g of soil in 10 mL of 0.9% NaCl solution, from which 100 µL amounts were spread on the surface of fungus-selective medium (5 g L^−1^ glucose, 5 g L^−1^ KH_2_PO_4_, 1 g L^−1^ yeast extract, 20 g L^−1^ agar, 200 µg L^−1^ oxytetracyclin and 200 µg L^−1^ streptomycin (Sigma Aldrich, Schnelldorf, Germany). After 96 h of incubation at 25 °C, the growing *Trichoderma* colonies were inoculated to new Petri plates filled with the same medium for purification. Species-level identification of the *Trichoderma* isolates based on the sequence analysis of a fragment of the translation elongation factor 1 alpha (*tef1α*) gene amplified with the EF1-728F (5’-CATCGAGAAGTTCGAGAAGG-3’) and TEF-LLErev (5’-AACTTGCAGGCAATGTGG-3’) primers, and a fragment of the RNA polymerase (*rpb2*) gene amplified with the fRPB2-5F (5’-GAYGAYMGWGATCAYTTYGG-3’) and fRPB2-7cR (5’-CCCATRGCTTGYTTRCCCAT-3’) primers were performed as described earlier [9,29]. Isolation of *B. velezensis* strains and their identification based on the sequence of the *gyrA* and *rpoB* marker regions as well as fatty acid profiling were performed in a previous study by Huynh et al. [26]. Strain *B. velezensis* SZMC 25003 and the *B. mojavensis* strains were identified by the sequence analysis of a fragment of the DNA gyrase alpha subunit (*gyrA*) amplified with the GyrA-F (5′-CAGTCAGGAAATGCGTACGTCCTT-3′) and GyrA-R (5′-CAAGGTAATGCTCCAGGCATTGCT-3′) primers as described previously [30]. For the isolation of potentially nitrogen-fixing bacteria, 1 g soil was suspended in 10 mL of 0.9% NaCl solution and a four-step 10× dilution series was prepared. From the dilution steps, 50–50 µL amounts were spread on the surface of nitrogen source free NB medium (5 g L^−1^ glucose, 5 g L^−1^ mannitol, 0.1 g L^−1^ CaCl_2_, 0.1 g L^−1^ MgSO_4_, 0.005 g L^−1^ Na_2_MoO_4_, 0.9 g L^−1^ K_2_HPO_4_, 0.1 g L^−1^ KH_2_PO_4_, 0.01 g L^−1^ FeSO_4_, 5 g L^−1^ CaCO_3_, 20 g L^−1^ agar). The growing bacterial colonies were isolated after two weeks of incubation at 25 °C. The identification was performed based on the sequence analysis of a fragment of the 16S RNA gene amplified by the Eub-8F (5′-AGAGTTTGATCCTGGCTCAG-3′) and Eub-534R (5′-ATTACCGCGGCTGCTGG-3′) primers [31]. Isolation of the glyphosate-tolerant *Ensifer adherens* and *Pseudomonas resinovorans* strains and their identification was described earlier [27].

### 2.2. Measurements of Extracellular Enzyme Activities of Trichoderma Strains

The cellobiohydrolase, β-glucosidase, β-xylosidase and phosphatase enzyme activity measurements of *Trichoderma* strains were performed according to Chen et al. [32].

For the measurement of the N-acetyl-β-D-glucosaminidase activity, 1.5 g chitin from shrimp shells (Sigma Aldrich, Schnelldorf, Germany) was spread into empty Petri plates, which were then sterilized and inoculated with a total amount of 2 × 10^6^ conidia. After 168 h of incubation at 25 °C, 25 mL of distilled water was added to the cultures and the dishes were placed to 4 °C for 2 h. At the end of the cold treatment, 2 mL solutions were placed into Eppendorf tubes and stored at −20 °C. N-acetyl-β-D-glucosaminidase activities were measured according to Chen et al. [32] with the chromogenic substrate 4-nitrophenyl-N-acetyl-β-D-glucosaminide (Sigma Aldrich, Schnelldorf, Germany). 

The U mL^−1^ values of enzyme activities were calculated by the following equation: ((A/ε × l) × 10^6^)/60, where “A” is the absorbance of the solution at 405 nm, “ε” is the molar extinction coefficient (for p-nitrophenol: 1.75 × 10^4^ M^−1^ cm^−1^) and “l” is the pathlength of the light in the solution. All measurements were carried out in three biological replicates.

### 2.3. Measurement of Fungicide Tolerance of Trichoderma Strains

The sensitivity of *Trichoderma* strains to fungicides was tested using microdilution method on 96-well microtiter plates (Sarstedt, Nümbrecht, Germany). Eight fungicides (carboxin, bromoxynil, thiram, amitraz, cyproconazole, folpet, imazalil and penconazole, Sigma-Aldrich, Budapest, Hungary) were tested at the concentrations of 250, 125, 62.5, 31.25, 15.625, 7.8125, and 3.9625 μg mL^−1^. One-one mg of each fungicide was first dissolved in 100 μL distilled water, then added to YEG (2 g L^−1^ yeast extract, 2 g L^−1^ glucose) medium in the appropriate amount. Two-fold dilution series were prepared from the fungicide solutions in the wells of 96-well microtiter plates. The final volume in the wells was 200 μL, which was then inoculated with an amount of 2×10^6^ conidia/well, resulting in a final concentration of 10^7^ conidia mL^−1^, which was between the concentration values used for soaking sweet potato cuttings and for secondary treatment (10^8^ and 10^6^ conidia mL^−1^, respectively, see Section 2.9). For the determination of fungicide sensitivity, the optical density (OD) of the samples was measured at a wavelength of 620 nm using a Spectrostar Nano microtiter plate reader (BMG Labtech, Ortenberg, Germany). Measurements took place in three biological replicates both immediately after inoculation and after 72 h of incubation at 25 °C. Minimum inhibitory concentration (MIC) values were calculated using the Microsoft Excel 2010 software. The data from the immediate measurements were subtracted from the results of the 72 h measurements, the resulting value was multiplied by 100 and then divided by the average values determined for three samples of the growth control in YEG. The effect of the fungicides was considered inhibitory if the growth of *Trichoderma* did not exceed 20% of the growth of the positive control.

### 2.4. Measurement of the Effect of pH, Temperature and Water Activity on the Growth of Trichoderma Strains

Measurements of the effect of pH (pH values of 5, 6, 7, and 8 set by the addition of Britton-Robinson universal buffer [33]), temperature (25, 30, 35 and 40 °C) and water activity (a_w_ values of 0.998, 0.994, 0.989, 0.983, 0.970 and 0.957 set by the addition of NaCl according to Resnik and Chirife [34]) on the mycelial growth of *Trichoderma* strains were performed on PDA (potato dextrose agar, 24 g L^−1^, VWR, Debrecen, Hungary) medium inoculated with 5 mm agar disks cut from the edge of 48 h-old PDA-grown colonies. The test was conducted in three biological replicates and the colony diameters were measured every 24 h for three days in the case of the water activity and temperature tests, and for four days in the case of the pH test.

### 2.5. In Vitro Antagonistic Test of Trichoderma Strains

The evaluation of Biocontrol Index (BCI) values of *Trichoderma* strains followed the protocol described by Szekeres et al. [35]. Briefly, the *Trichoderma* strains and the fungi known as plant pathogens were confronted on the surface of yeast extract—glucose medium, and the BCI values were determined after image analysis of the photographs taken from the plates 10 days after the inoculation of *Trichoderma* according to the formula: BCI = (area of the *Trichoderma* colony / total area occupied by the colonies of both *Trichoderma* and the plant pathogenic fungus) × 100. Nine strains of fungal species known as plant pathogens from the Szeged Microbiology Collection (*Fusarium solani* SZMC 16084, *Botrytis cinerea* SZMC 21047, *Colletotrichum gloeosporioides* SZMC 16086, *Sclerotinia sclerotiorum* SZMC 6250J, *Alternaria solani* SZMC 6241J, *Fusarium culmorum* SZMC 11039, *Fusarium graminearum* SZMC 11030, *Gaeumannomyces graminis* SZMC 23658 and *Phoma cucurbitacearum* SZMC 16088) were tested in the in vitro antagonism experiments in three biological replicates.

### 2.6. Antagonisms Tests of Bacillus Strains against Strains of Fungal Species Known as Plant Pathogens

In vitro antagonism tests of *Bacillus* strains were performed on PDA medium at 25 °C. The tested fungi were those listed in Section 2.5. Disks 6 mm in diameter from three to five day old fungal colonies grown on PDA were used in the tests. The tested bacteria were inoculated onto the PDA plates 25 mm from the inoculation point of the fungus. The incubation lasted until the fungal colony radius reached 25 mm on the control plates that were not inoculated with any bacterial strains. After the incubation, the colony radius of the plant-pathogenic fungus and the distance between the fungal and bacterial colonies were measured in three biological replicates.

### 2.7. Evaluation of Growth of Potentially N_2_-Fixing Bacteria in Nitrogen Source Free Medium

The growth parameters of the isolated candidate nitrogen-fixing bacteria were tested on NB medium. Overnight bacterial cultures grown in YEG medium were washed three times with 9 g L^−1^ NaCl solution and then suspended in 9 g L^−1^ NaCl. Ten µL of the suspensions were inoculated to NB medium and the growth of the strains followed for 96 h at 25 °C. The colonies were visually evaluated, and the following categories were defined: poor growth, moderate growth, substantial growth.

### 2.8. Characterization of Bacterial Isolates

The modified broth microdilution assay (serial dilution) described by Vörös et al. [36] was used to study the effect of 18 herbicides (bensulfuron-methyl, chlorotoluron, chlorpropham, chlorsulfuron, cinosulfuron, diuron, dimethachlor, fenuron, ethoxysulfuron, glyphosate (AK Scientific, Union City, CA, USA), *Glialka Star* (Monsanto, St. Louis, MO, USA), isoproturon, linuron, 2-methyl-4-chlorophenoxyacetic acid (MCPA), primisulfuron-methyl, propham, triasulfuron and 2,4-dichlorophenoxyacetic acid (2,4-D) (Sigma-Aldrich, Budapest, Hungary), 14 fungicides (captan, carbendazim, carboxin, fenarimol, flutriafol, imazalil, mancozeb, maneb, penconazole, tebuconazole, thiabendazole, thiram, thiophanate-methyl and zineb) (Sigma-Aldrich, Budapest, Hungary) as well as the insecticide diflubenzuron (Sigma-Aldrich, Budapest, Hungary) on bacterial growth. All pesticides were dissolved in 96% ethanol at 0.25 mg mL^−1^, with the exception of glyphosate which was dissolved in distilled water. Bacterial strains were incubated in 100 μL PDB amended with the tested pesticide at concentrations of 25, 12.5, and 6.25 μg mL^−1^ in 96-well microtiter plates. For inoculation, bacterial suspensions of 10^5^ CFU mL^−1^ were prepared from overnight cultures in 5 mL physiological saline solution and this suspension was used to inoculate the well to a starting concentration of 2 × 10^4^ CFU mL^−1^. Inoculated PDB without pesticide and non-inoculated PDB amended with the corresponding pesticide served as positive and negative controls, respectively. Cell density (OD_620_) was determined spectrophotometrically with a Spectrostar Nano microtiter plate reader after 48 h of incubation at 25 °C. All tests were performed on three biological replicates.

The method reported by Patel et al. [37] in a modified form [27] was applied to study bacterial salinity tolerance using broth microdilution assay in 96-well microtiter plates. The drought tolerance test was carried out according to Devi et al. [38] with modifications. PEG 6000 concentrations were 250, 125, 62.5, 31.3, 15.6, 7.8, and 3.9 g L^−1^ in PDB (potato dextrose broth, VWR, Debrecen, Hungary), corresponding with osmotic potential values of −0.74, −0.22, −0.07, −0.03, −0.01, −0.005, and −0.002 MPa [39], respectively. Optical densities of the cultures were measured at 620 nm with a Spectrostar Nano microtiter plate reader in three biological replicates.

Determination of the pH range for bacterial growth was studied in YEG medium adjusted with Britton-Robinson buffer [33]. Target pH values (6.09, 6.59, 7.00, 7.54, 7.96, 8.36 and 8.95) were obtained by mixing 100 mL solution containing 0.04 M CH_3_COOH, 0.04 M H_3_PO4 and 0.04 M H_3_BO_3_ with the defined amounts of 0.2 M NaOH (42.5, 47.5, 52.5, 57.5, 60.0, 62.5 and 67.5 mL, respectively). YEG media with the adjusted pH values were inoculated to 10^5^ CFU mL^−1^ as the initial cell concentration using overnight bacterial cultures grown in 5 mL PDB. The optical densities were measured at 620 nm with a Spectrostar Nano microplate reader after five days of incubation at 25 °C. Buffers and YEG were prepared at double concentrations and mixed in equal ratios. The tests were carried out in three biological replicates.

IAA-producing abilities of glyphosate-tolerant bacterial isolates were screened based on the method of Shrivastava and Kumar [40] with minor modifications. PDA plates were amended with L-tryptophan, the precursor of IAA at 1 g L^−1^ and holes 1 cm in diameter were made with a sterile cork-borer. The holes were filled with 200 μL of bacterial culture pregrown overnight in 30 mL PDB and the plates were incubated for 48 h at 25 °C. After incubation, the growing bacterial cultures were removed from the medium surface with tissue paper and each hole was filled with 200 μL Salkowski reagent (12 g L^−1^ FeCl_3_ dissolved in 37% H_2_SO_4_ [41]). HPLC measurements were performed as described by Zhumakayev et al. [27].

### 2.9. Production of Microbial Inoculum for Field Experiments

*Trichoderma ghanense* SZMC 25217 and *Trichoderma afroharzianum* SZMC 25231 were cultured on PDA medium at room temperature for seven days. After the incubation time, the conidia were washed off from the surface of the medium and 1 L suspensions of 2 × 10^6^ conidia mL^−1^ were made of each strain in 9 g L^−1^ NaCl solution. The bacterial strains *Bacillus velezensis* SZMC 24986, *Arthrobacter globiformis* SZMC 25081, and *Pseudomonas resinovorans* SZMC 25872 were cultured in PDB medium. At first, 1 mL PDB was inoculated with microstreaker, with each strain separately in test tubes. After shaking the cultures at 140 rpm overnight at 25 °C, they were used to inoculate 50 mL PDB media in 250 mL Erlenmeyer flasks, that were shaken at 140 rpm overnight at 25 °C. The grown cultures were used to inoculate 1 L of PDB media and shaken at 140 rpm for 48 h at 25 °C. The concentration of the resulting cultures of microorganisms was adjusted to 5 × 10^8^ conidia mL^−1^ for both selected *Trichoderma* components and 5 × 10^8^ cells mL^−1^ for each of the three selected bacteria. The components were mixed in a proportion of 1:1:1:1:1 (resulting in a mixture containing 10^8^ conidia/cells mL^−1^ for each component), which was then used for soaking the sweet potato secondary cuttings. For the application of secondary sweet potato treatment on the field, the mixture was diluted 1:100 in water.

### 2.10. Field Experiments

Two field experiments were performed in consecutive years (2019 and 2020). In both years, the registered Hungarian sweet potato variety ’Ásotthalmi12′ of orange flesh and reddish/tan skin was used.

In 2019, the experiment was carried out in Szentes, South-East Hungary, on slight alkaline (pH 7.3) clay-loam soil of average humus (2.01%), extreme AL-soluble P_2_O_5_ (2249 mg kg^−1^) and very good AL-soluble K_2_O (471 mg kg^−1^) content. The preceding crop was pepper, with no manure, fertilizer, and soil disinfection applied. The irrigation was performed via a sprinkler system. Sweet potato secondary cuttings were planted in flat, with 0.8 m row spacing and 0.2 m plant-to-plant distance on 27 June 2019. Each plot consisted of 30 plants distributed in two rows of 3 m in length. The experimental setup was randomized block with four replications. There were four treatments applied: Treatment 1 (T1): soaking the basic part of sweet potato secondary cuttings for 10 min in the mixed microbial suspension. Treatment 2 (T2): the same as Treatment 1 with an additional inoculation of the soil with the mixed microbial suspension at a dosage of 0.15 L per plant near the sweet potato rows on the 34th day after planting. Treatment 3 (T3): inoculation of the soil with the mixed microbial suspension near the sweet potato rows before planting at a dosage of 0.15 L per plant. Treatment 4 (T4): the same as Treatment 3 with an additional inoculation of the soil with the mixed microbial suspension at a dosage of 0.15 L per plant near the sweet potato rows on the 34th day after planting. Control (C): no microbial treatments applied. Storage roots were harvested on 15 October 2019. Total storage root weight per plot was determined and divided by the number of surviving plants per plot (determined before harvest by counting the plants one-by-one: 26–30 plants per plot) to get the average storage root weight per plant in the given replication of the relevant treatment. The average weight of storage roots was determined for each treatment based on the average weight of each 10th–15th randomly chosen storage root in each treatment and replication.

In 2020, the experiment was carried out in Ásotthalom, South-East Hungary, on alkaline (pH 7.78) sandy soil of poor humus (0.43%), very good AL-soluble P_2_O_5_ (307 mg kg^−1^) and poor AL-soluble K_2_O (69.6 mg kg^−1^) content. The preceding crop was sweet potato. No soil disinfection was applied. Sweet potato secondary cuttings were planted in flat, in a twin-row system on 5 June 2020. The row spacing was 0.4 m and 0.8 m between the twin rows and the two-row blocks, respectively. The plant-to-plant distance was 0.3 m. One twin-row block of two rows consisted of 360 plants. Each basic treatment (with/without fertigation) covered one block. The fertigation was applied according to a usual farm-size technology, adding K-stressed fertilizer complex into the drip irrigation system. In the fertigation-free basic treatment, the irrigation system was locked during the addition of the fertilizer. The secondary treatments were applied for 120 plants each. The following treatments were applied: Treatment 1: soaking the basic part of sweet potato secondary cuttings for 10 min in the mixed microbial suspension before planting, planting in the fertilized unit. Treatment 2: the same preparation of cuttings as in Treatment 1, planting in the non-fertilized unit. Treatment 3: the same as Treatment 1 with an additional inoculation of the soil with the mixed microbial suspension at a dosage of 0.15 L per plant near the sweet potato rows on the 30th day after planting. Treatment 4: the same as Treatment 2 with an additional inoculation of the soil with the mixed microbial suspension as described for Treatment 3. Control 1: no microbial treatments applied, planting in the fertilized unit. Control 2: no microbial treatments applied, planting in the non-fertilized unit. Storage roots were harvested on 20 October 2020. Each block of the treatments was divided into four parts to form virtual replication plots. The total yield of a virtual plot was divided by the number of surviving plants per plot (determined before harvest by counting the plants one-by-one: ca. 30 plants per plot) to obtain the average storage root weight per plant.

### 2.11. Statistical Analysis

The experimental data were statistically evaluated using Microsoft Excel 2013 and SPSS for Windows 13.0. For the evaluation of the results from the in vitro experiments, standard deviation (SD) values were calculated, and Student’s t-test was performed. In the case of the field experiment in 2019, one-way analysis of variance (ANOVA) was used for the statistical analysis of the results. Regarding the data proven to be significant by ANOVA, the averages of the factors were compared by the LSD as well as the Tukey test at the probability levels of 5% and 1%.

## 3. Results

### 3.1. Selection of Microorganisms

#### 3.1.1. Characterization of *Trichoderma* Strains

The *tef1α* sequence-based identification revealed that the isolates are belonging to five *Trichoderma* species: *T. afroharzianum*, *T. ghanense*, *T. guizhouense*, *T. harzianum* and *T. virens* (Table 1).

**Figure 1:**
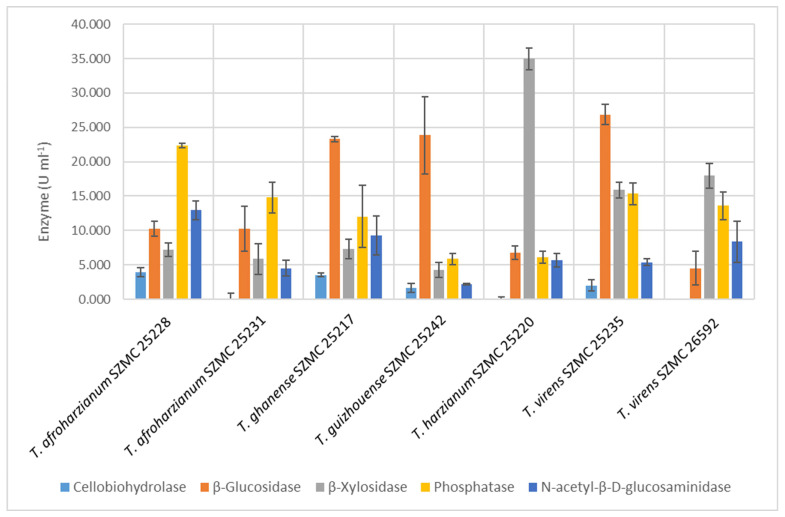
Extracellular enzyme activity values of 7 tested *Trichoderma* strains (mean ± SD, *n* = 3).

The isolates were tested for their ability to produce cellulose-, xylan-, and chitin-degrading, as well as phosphorous-mobilizing extracellular enzymes (Figure 1). In general, the cellobiohydrolase activity values were much lower than in the case of the other enzymes. Most of the strains that showed an enzyme activity peak for one of the five tested enzymes also showed inferior activity in the other tests. For example, the β-xylosidase activity of *Trichoderma harzianum* SZMC 25220 was one of the highest, but it had no cellobiohydrolase and only low levels of β-glucosidase and phosphatase activities. On the other hand, there were several strains that showed average activities for most of the tested enzymes, and some of them even showed high activity values of one of the enzymes. In this regard the performance of strains *T. ghanense* SZMC 25217, *T. virens* SZMC 25235, and *T. afroharzianum* SZMC 25228 was the best (Figure 1). Strains *T. ghanense* SZMC 25217, *T. guizhouense* SZMC 25242, and *T. virens* SZMC 25235 were outstanding in β-glucosidase activity. Strain *T. afroharzianum* SZMC 25228 was exceptional in N-acetyl-β-D-glucosaminidase enzyme activity with over 12 U mL^−1^, followed by the strains *T. ghanense* SZMC 25217 and *T. virens* SZMC 26592 with 9.25 U mL^−1^ and 8.34 U mL^−1^, respectively.

The application potential of the *Trichoderma* strains in combination with pesticides is a possible strategy within the frames of integrated pest management, therefore the sensitivity of *Trichoderma* strains to fungicides was tested. The strains also showed high tolerance to a set of the tested fungicides (Table 2). The MIC values of some antifungal agents were the highest or close to the highest concentrations tested (250 or 125 µg mL^−1^). Amitraz and penconazole were effective against some, while imazalil against most of the *Trichoderma* strains. The MIC values of imazalil were 15.625 µg mL^−1^ for *T. guizhouense* SZMC 25242, 7.8125 µg mL^−1^ for *T. afroharzianum* SZMC 25228 and *T. afroharzianum* SZMC 25231, and 3.9625 µg mL^−1^ for *T. virens* SZMC 26592 and *T. harzianum* SZMC 25220. Strains *T. ghanense* SZMC 25217 and *T. virens* SZMC 25235 showed the best tolerance properties for the tested fungicides, tolerating even the most effective fungicide imazalil with the MIC value of 62.5 µg mL^−1^.

**Figure 2:**
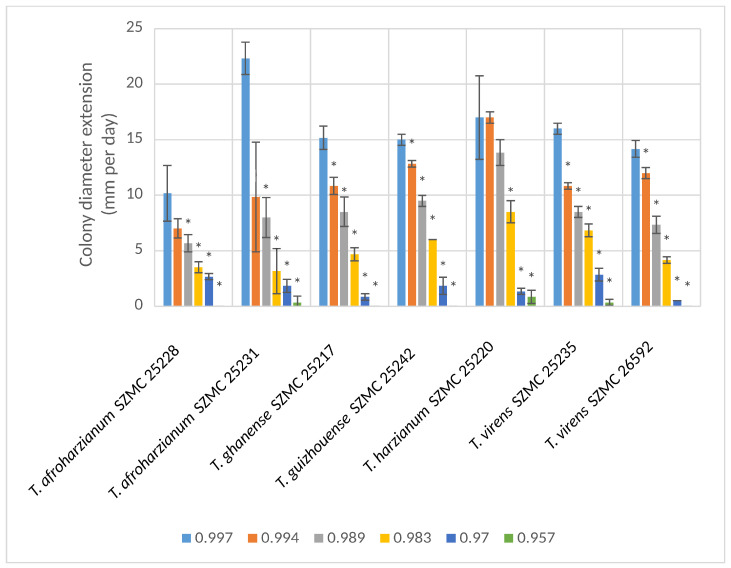
Colony diameter extension rates of the tested *Trichoderma* strains at different water activity values (mean ± SD, *n* = 3). Asterisks (*) indicate statistically significant (*p* ≤ 0.05) differences in relation to the colony diameter extension rate of the respective strain at the water activity optimum (a_w_ 0.997).

The tolerance of the strains to low water availability was also examined. At or below a_w_ 0.983, the growth of all tested strains was inhibited compared to the NaCl-free control (a_w_ 0.997), and below 0.970 their colony diameter extension rate was only at or below 4.78 mm day^−1^. At the water activity values of 0.983 and 0.970, the strain *T. harzianum* SZMC 25220 showed the best growth ability with a colony diameter extension rate of 3.1 mm day^−1^ (Figure 2).

**Figure 3:**
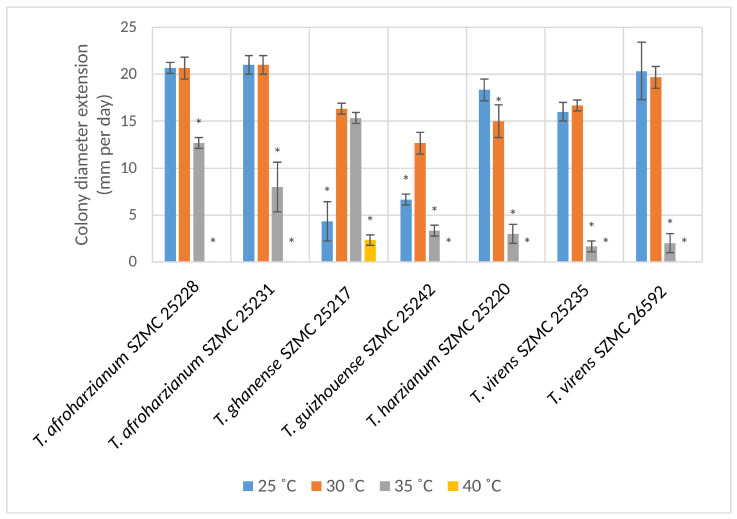
Colony diameter extension rates of the tested *Trichoderma* strains at different temperatures (mean ± SD, *n* = 3). Asterisks (*) indicate statistically significant (*p* ≤ 0.05) differences in relation to the colony diameter extension rate of the respective strain at the optimum temperature.

All tested strains grew well and similar to each other both at 25 °C and 30 °C (Figure 3). Strain *T. ghanense* SZMC 25217 overgrew the Petri plates by the end of the 72 h incubation with a colony diameter extension of 28 mm per day. The strain was able to overgrow the entire Petri plate in 72 h even at 35 °C, while the growth rate of all the other strains was much slower and strains *T. harzianum* SZMC 25220 and *T. virens* SZMC 25235 could not grow at this temperature. Only strain *T. ghanense* SZMC 25217 was able to grow at 40 °C.

**Figure 4:**
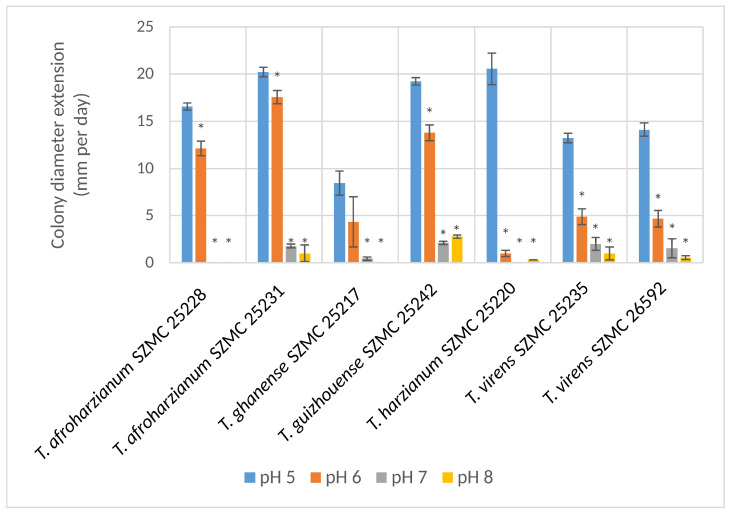
Colony diameter extension rates of the tested *Trichoderma* strains at different pH values (mean ± SD, *n* = 3). Asterisks (*) indicate statistically significant (*p* ≤ 0.05) differences in relation to the colony diameter extension rate of the respective strain at pH 5.

All strains grew well at pH 5, while most of them also at pH 6 (Figure 4), except for strains *T. virens* SZMC 25235, SZMC 26592 and *T. ghanense* SZMC 25217, that grew slower at pH 6, while the colony diameter extension of strain *T. harzianum* SZMC 25220 was only 3 mm per day. At pH 7 and 8, some strains grew at a slow colony diameter extension rate, while strains *T. harzianum* SZMC 25220 and *T. afroharzianum* SZMC 25228 could not grow. Strains *T. afroharzianum* SZMC 25231, *T. atroviride* SZMC 26594, *T. virens* SZMC 25235, SZMC 26594, *T. ghanense* SZMC 25217, SZMC 26850 and *T. guizhouense* SZMC 25242 could grow at pH 7 and only strain *T. guizhouense* SZMC 25242 grew at pH 8 with a slow colony diameter extension of 4.9 mm per day.

**Figure 5:**
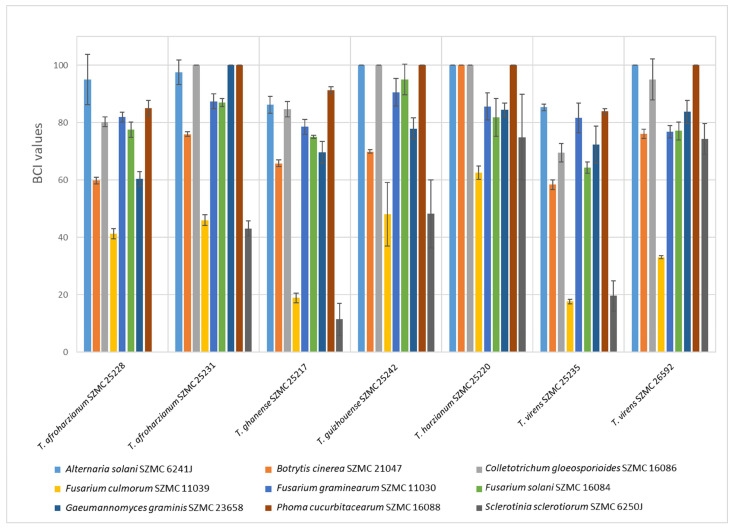
In vitro biocontrol index (BCI) values of the tested *Trichoderma* strains towards strains of fungal species known as plant pathogens (mean ± SD, *n* = 3). Higher values refer to more intensive overgrowth of the plant pathogenic fungus by the *Trichoderma* strain. A BCI value of 100 means complete overgrowth.

During the in vitro confrontation tests, most of the *Trichoderma* strains performed well against *Colletotrichum gloeosporioides* SZMC 16086 and *A. solani* SZMC 6241J (Figure 5). Strain *S. sclerotiorum* SZMC 6250J tolerated the presence of most of the *Trichoderma* strains with the exception of *T. harzianum* SZMC 25220 and *T. virens* SZMC 26592.

#### 3.1.2. Selection of Antagonistic and Potentially N-Fixing Bacteria

**Figure 6:**
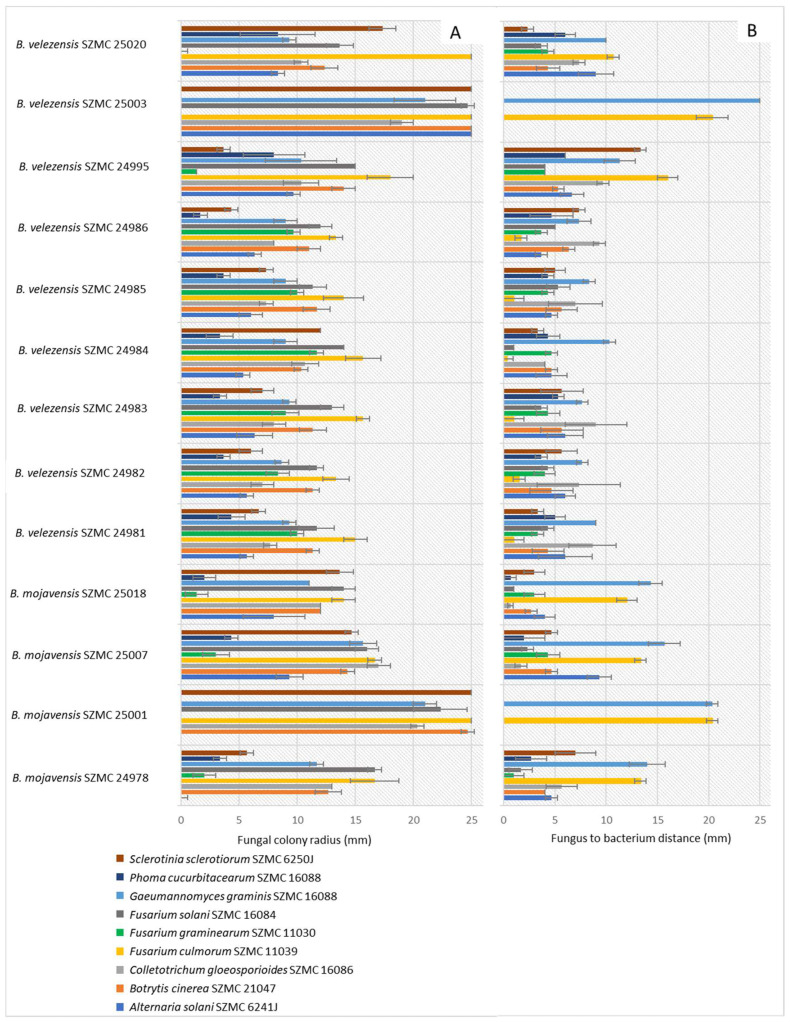
In vitro antagonism of *Bacillus* strains towards strains of fungal species known as plant pathogens. (**A**). Colony radius of the tested fungi in the presence of biocontrol candidate *Bacillus* strains. (**B**). Distance between fungal and *Bacillus* colonies (mean ± SD).

Thirteen *Bacillus* strains were isolated for biocontrol purposes from different soil samples collected from agricultural fields in Hungary and Serbia. During the in vitro antagonism tests, the bacterial strain resulting in the largest distance to the strains of fungal species known as plant pathogens and their smallest colony radius was selected. The most efficient strain was *Bacillus velezensis* SZMC 24986 (Figure 6).

Twenty potentially N_2_-fixing bacterial strains were isolated (Table 1). *Arthrobacter globiformis* SZMC 25081 has formed the largest colony on nitrogen source free medium (Table 3). This strain was selected for the soil inoculant.

#### 3.1.3. Indole-3-Acetic Acid Production and Stress Tolerance of Bacterial Isolates

**Figure 7:**
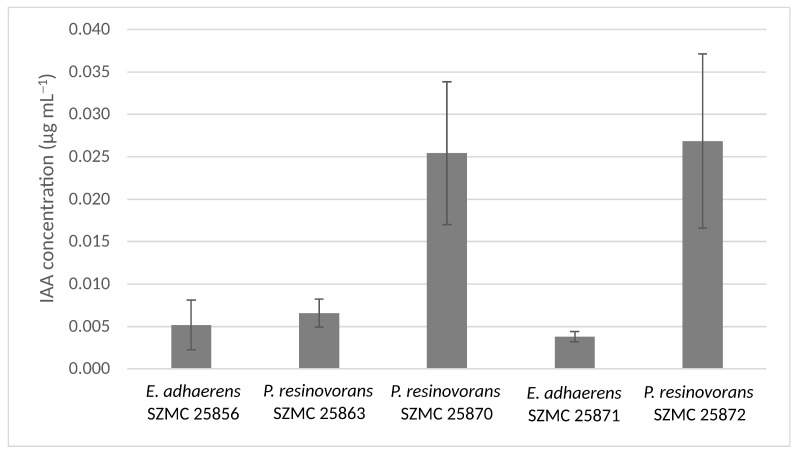
Indole-3-acetic acid (IAA) production of potential plant growth promoting glyphosate-tolerant bacterial strains measurep by HPLC (mean ± SD).

The IAA production of strains *P. resinovorans* SZMC 25870 and SZMC 25872 were significantly higher compared to the other examined strains (*p* ≤ 0.05, Student’s *t*-test) (Figure 7). The IAA concentration in the culture supernatant of strain *P. resinovorans* SZMC 25872 was 0.027 µg mL^−1^ in the absence of tryptophan, which proved to be the highest among the examined glyphosate-tolerant bacterial isolates, therefore this strain was selected for the purposes of plant growth promotion and further characterized.

We tested the tolerance of strain *P. resinovorans* SZMC 25872 to stress factors. The favorable pH range of this strain was found in the neutral and slightly alkaline range between 7.00 and 8.36. Assays for tolerance to adverse abiotic factors revealed that strain SZMC 25872 could tolerate salinity up to 6.3 g L^−1^ NaCl and drought up to 125 g L^−1^ PEG (−0.22 MPa), which may allow the utilization of this strain under drier conditions. Pesticide sensitivity assays revealed tolerance of SZMC 25872 to the majority of the studied pesticides up to 25 µg mL^−1^. Among the studied pesticides, only fungicides, such as maneb mancozeb inhibited SZMC 25872 at the lowest studied concentration (6.25 µg mL^−1^), while it could tolerate 12.5 µg mL^−1^ of the herbicide ethoxysulfuron, as well as 6.25 µg mL^−1^ of captan, thiram and zineb (Table 4).

#### 3.1.4. Selection of Microbial Components for a Soil Inoculant

Based on the data gathered, we selected the strain *T. ghanense* SZMC 25217 for its high enzyme activity values especially for cellulose-degrading enzymes. Strain *T. afroharzianum* SZMC 25231 was selected for biocontrol purposes based on its good in vitro antagonistic abilities towards strains of fungal species known as plant pathogens. Both selected *Trichoderma* strains could tolerate the presence of the tested fungicides, suggesting that they may also be applied in combination with pesticides within the frames of Integrated Pest Management (IPM). The performance of *B. velezensis* SZMC 24986 in in vitro antagonism tests against potentially plant pathogenic fungi makes it another promising candidate for antifungal biocontrol. The *A. globiformis* SZMC 25081 strain showed the strongest growth on nitrogen free medium which suggests possible nitrogen fixation, therefore we selected this strain for its potential to increase nitrogen availability for the plants. The IAA production of *P. resinovorans* SZMC 25872 may potentially result in plant growth promotion. The cultures of these selected microorganisms were prepared for the field experiments as described in Section 2.11 of the Materials and Methods. 

### 3.2. Performance of the Assembled Soil Inoculant in Sweet Potato Cultivation

**Figure 8:**
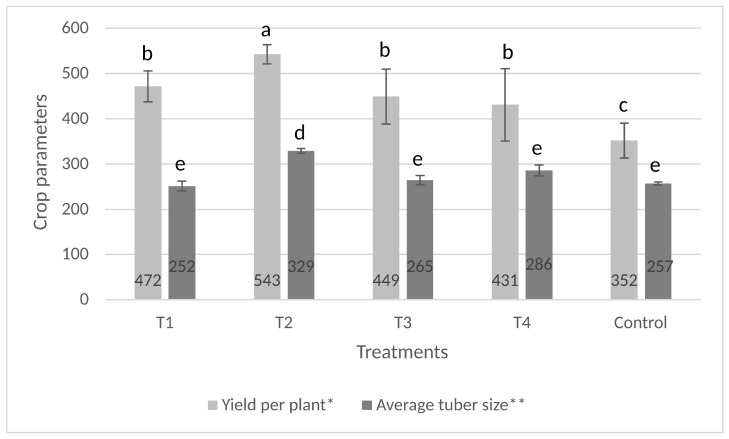
Effect of microbial treatment on sweet potato storage root yield and average tuber size at Szentes in 2019. The same letters above the data in the columns mean no significant difference (*p* ≥ 0.05) (mean ± SD) by the LSD test * or Tukey test **. T1: soaking secondary cuttings in the mixed microbial suspension; T2: same as T1 with an additional inoculation of the soil with the mixed microbial suspension; T3: inoculation of the soil with the mixed microbial suspension near the rows before planting; T4: same as T3 with an additional inoculation of the soil with the mixed microbial suspension; C: control (no microbial treatments applied).

**Figure 9:**
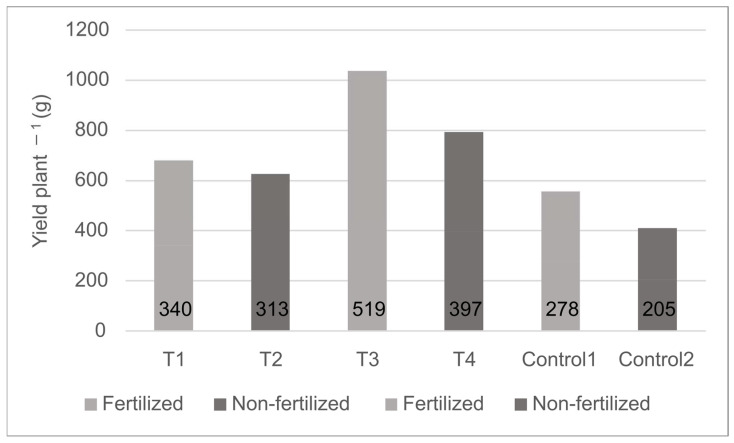
Effect of microbial treatment on the performance of sweet potato storage root yield per plant at Ásotthalom in 2020. T1: soaking sweet potato cuttings in the mixed microbial suspension before planting in the fertilized unit; T2: same preparation of cuttings as in T1 with planting in the non-fertilized unit; T3: same as T1 with an additional inoculation of the soil; T4: same as T2 with an additional inoculation of the soil; Control 1: no microbial treatments applied, planting in the fertilized unit; Control 2: no microbial treatments applied, planting in the non-fertilized unit.

In 2019, the differences between the treatment mean yields were not significant according to ANOVA. The LSD analysis, however, could show significant difference (*p* ≤ 0.05) between Treatment 2 (soaked cuttings + second treatment) and the untreated control (Figure 8). The microbial treatments had a positive effect, resulting in a higher storage root yield per plant (431–542.5 g) compared to that of the control (352 g). Inoculation of soil with the microbial complex five weeks after planting increased the yield in the case of soaked cuttings, thus resulting in the highest yield (542.5 g) in the experiment. The second treatment slightly decreased the yield if the cuttings were planted into inoculated soil, but the difference was not significant and could have resulted from the poorer performance in one of the replications (Figure 9). Calculating with the experimental setup of 62.500 plants ha^−1^, the difference between the treated (26.9–33.9 tons ha^−1^) and the control (22 tons ha^−1^) plots can be between 4.9 and 11.9 tons at the hectare level.

The highest average storage root size was also achieved in the treatment of soaked cuttings plus second treatment. The 329 g was significantly different from the results achieved in all other treatments including the control (251–286 g).

In 2020, the highest results were achieved with the double treatments (T3 and T4, basic + second treatment), followed by the basic only treatments (T1 and T2) (Figure 9). The results in the fertigated plots were higher than the respective, non-fertigated plots. It is remarkable that both microbial treatments in the non-fertigated blocks (T2 and T4, 313 g and 397 g, respectively) resulted in higher yields than the fertigated control (Control 1) representing the conventional technology applied on the farm (278 g) (Figure 9). Calculating with the experimental setup of 55.556 plants ha^−1^, the difference between the treated (17.4–28.8 tons ha^−1^) and control (11.4–15.4 tons ha^−1^) plants can be between 6.0 and 13.4 tons at the hectare level.

## 4. Discussion

Based on the in vitro studies, five microorganisms were selected to a potential agricultural soil inoculant: *Trichoderma ghanense* SZMC 25217, *Trichoderma afroharzianum* SZMC 25231, *Bacillus velezensis* SZMC 24986, *Arthrobacter globiformis* SZMC 25081 and *Pseudomonas resinovorans* SZMC 25872. The *T. ghanense* SZMC 25217 was selected for polysaccharide degradation and phosphorous mobilization based on its wide range of relatively high enzyme activity values, including cellulolytic, xylanolytic and chitinolytic activities as well as phosphatase. Strain *T. afroharzianum* SZMC 25231 showed promising in vitro antagonism against strains of fungal species known as plant pathogens and also grew at higher pH values, which may contribute to its survival under different soil conditions. We selected this strain as a potential biocontrol component of the inoculant. The potentially plant pathogenic fungi involved in the in vitro antagonism test were selected based on literature data. Several *F. solani* isolates were recovered from *Ipomoea batatas* affected by root rot [42], and *S. sclerotiorum* was also isolated from sweet potato showing white rot symptoms [43]. Grey mold of sweet potato tubers during storage caused by *Botrytis cinerea* [44], leaf and stem blight caused by *A. solani* [45], and anthracnose caused by *Colletotrichum* [46] are known from the literature. We also evaluated the BCI values against further filamentous fungi that are not known as pathogens of sweet potato to investigate the potential applicability of the selected strains against pathogens of other crops. *Trichoderma* strains are well known for their ability to produce high amounts of extracellular enzymes [12,47,48,49]. They are efficient competitors of other microorganisms in the soil and eventually they parasitize other filamentous fungi [6,50,51]. It is described that chitin-degrading enzyme activity eventually correlates with the biocontrol activities of several microorganisms [52,53].

Strain *B. velezensis* SZMC 24986 showed promising in vitro antagonism against all of the tested strains of fungal species known as plant pathogens. The antibacterial and antifungal effects of this species were described earlier [54,55,56], which supports this strain as a potential candidate for biocontrol purposes. Nitrogen-fixing bacteria play an important role in the soil, therefore they are becoming important parts of multicomponent soil inoculants [57]. The ability of certain *Arthrobacter* strains to fix nitrogen is known from the literature [58]. The selected *A. globiformis* strain SZMC 25081 showed promising growth on nitrogen-free medium compared to the other candidates. The production of IAA by a microorganism used to correlate with its plant growth promoting ability [59], therefore we also selected an IAA-producing *P. resinovorans* strain, SZMC 25872. The genus *Pseudomonas* is well known for IAA production [60,61,62] and several strains were also tested on *Sedum alfredii* and *Arabidopsis thaliana* with promising results [63,64]. The genome sequence of the *P. resinovorans* strain SZMC 25872 [28] contains the tryptophane side chain oxidase (*tso*) and the aldehyde dehydrogenase (*AldA*) genes enabling IAA production [65], as well as the genes *pqqB*, *pqqC* and *pqqE* of the pyrroloquinoline quinine biosynthetic pathway, which are contributing to phosphate solubilization [66].

IPM is a widely used strategy in modern agriculture [67]. The results from the tests examining the tolerance of the selected biocontrol microorganisms to different types of agricultural pesticides suggest that they have the potential to be combined with chemical pesticides within the frames of IPM strategies.

To test the efficiency of the consortial soil inoculant, two field experiments were performed in sweet potato cultivation. The field experiments revealed that sweet potato production can be efficient both on hard and on loose soils. It is remarkable that—although in different years—the storage root yield could be higher on hard soil, even without fertilizer application. One possible reason is the high natural nutrient content of the soil providing nutrients more efficiently compared to the drip irrigation system on the sandy soil. Other explanation can be the higher activity level of microorganisms under the conditions of the hard soil. 

The microbial complex applied could increase the sweet potato yield compared to both the fertilized and non-fertilized controls. The rate of increase in yield, however, was different in the various treatments. The highest rate could be achieved on sandy soil without fertigation—the yield was 1.53–1.94 times higher compared to the control. On sandy soil with fertigation, the rates were slightly lower (1.22–1.87 times higher than the control). On hard soil without fertilization the increase rates were lower, between 1.34 and 1.54 times higher than the control. These results suggest that fertilization or higher basic nutrient levels of soil can decrease the efficiency of the microbial treatment. 

Increased storage root yield of sweet potato was received with four strains of plant growth promoting rhizobacteria (PGPR), (*Klebsiella* sp. UPMSP9, *Erwinia* sp. UPMSP10, *Azospirillum brasilense* SP7, *Bacillus sphaericus* UPMB10) on sandy soil in ridge planting if cuttings were soaked in the bacterial suspension prior to planting and secondary treatment was applied one month after planting [25]. The best result was achieved with *Klebsiella* and moderate N supply, suggesting the possible reduction of N fertilizer rates. Inoculation of soil with various strains of *Azotobacter* (*Azotobacter* sp. IBCB10 and *Azotobacter vinelandii* IBCB15) performed eight days after planting could contribute to the decrease of N fertilization [68]. The application of *Azospirillum brasilense* (TI-Sp-7, ATCC 35629) inoculum into the soil two, four and six weeks after planting increased the storage root yield, especially if applied together with moderate N supply [69]. The higher yields were accompanied by lower foliage yields, suggesting that the inoculant may enhance storage root growth at the expense of foliage growth. As a result of an experiment with a commercial PGPR product, OYK Farming Ace, containing one *Bacillus* sp. strain (LC590219), the inoculated strain was not detected, and significant plant-growth-promoting effects were not observed. The inoculation, however, changed the endophytic bacterial composition, and the changes differed between the sweet potato cultivars involved [70].

Calculating with 62,500 plants per hectare in our experiment of 2019, the average yields per hectare were 29.5, 33.9, 28.1, 26.9, 22.0 tons for Treatments 1–4 and Control, respectively. In 2020, the number of plants calculated per hectare was 55,556, thus the average yields per hectare were 18.9, 17.4, 28.8, 22.1, 15.4 and 11.4 tons for Treatments 1–4 as well as Controls 1–2, respectively. The considerable differences between yields achieved with the identical treatments (e.g., T1 vs. T2, T2 vs. T4, Control vs. Control 2 in 2019 and 2020, respectively) could have been primarily caused by the poorer nutrient content of the soil in the experiment of 2020. Other possible reasons could have been the different crop year and irrigation strategy. The treatments with the microbial mixture, however, turned out to be superior over both the untreated controls and even over the fertilized control on sandy soil. Although comprehensive official data about the sweet potato yield in Hungary and in Europe are not available for 2019 and 2020, earlier data from FAOSTAT (https://www.fao.org/faostat/en/#data/QCL, accessed on 27 March 2023) and from Hungarian integrators [71] make a comparison possible. The average yield for Greece, Italy, Portugal and Spain (the only countries registered for sweet potato production in Europe by FAOSTAT) in 2017 was between 24 and 29 tons, while the average yield for Hungary is 18–25 tons per hectare. Thus, the microbial mixture applied in our experiments has a great potential for application in sustainable, and even in ecological farming in Hungary both on sandy and on hard soils.

## 5. Conclusions

In this study we assembled and tested a microbial consortium (synthetic community) for the treatment of sweet potato, consisting of two fungal (*T. ghanense* SZMC 25217, *T. afroharzianum* SZMC 25231) and three bacterial strains (*B. velezensis* SZMC 24986, *Arthrobacter globiformis* SZMC 25081, *P. resinovorans* SZMC 25872), which were selected to potentially meet a series of criteria in the form of a synthetic community (biological control of plant pathogens, plant growth promotion by phosphorus mobilization and nitrogen supply, polysaccharide degradation). Field experiments in sweet potato cultivation with the assembled soil inoculant revealed that the average tuber size of the treated plants and the yield per plant were higher compared to the untreated controls with and without fertilization. In the case of the type of treatment, where the propagation material of sweet potato was soaked and a later inoculation was also executed, the average tuber size and the yield per plant were also significantly higher than in the case of the untreated control. Based on the results, we managed to develop a microbiological soil inoculant promoting sweet potato growth. To the best of our knowledge, this is the first report about the successful application of a fungal-bacterial consortium in sweet potato cultivation. The yield increase data suggest that the application of microbial mixtures consisting of both bacterial and fungal components is a promising strategy for the efficient biological production of sweet potato.

## Figures and Tables

**Table 1:** Isolation and identification details of the microorganisms examined during this study.

SZMC * Number	Taxon	Basis of Identification	Origin of Soil Sample	Cultivated Crop	Reference
**Bacteria**					
SZMC 25080	*Arthrobacter* *globiformis*	16S rDNA (OP964023)	Totovo Selo (SRB)	pepper	this study
SZMC 25081	*Arthrobacter* *globiformis*	16S rDNA (OP964024)	Totovo Selo (SRB)	pepper	this study
SZMC 25084	*Variovorax* *paradoxus*	16S rDNA (OP964025)	Čantavir (SRB)	pepper	this study
SZMC 25085	*Nocardia* sp.	16S rDNA (OP964026)	Čantavir (SRB)	pepper	this study
SZMC 25093	*Peribacillus* *simplex*	16S rDNA (OP964027)	Čantavir (SRB)	pepper	this study
SZMC 25094	*Mycobacterium* sp.	16S rDNA (OP964028)	Čantavir (SRB)	tomato	this study
SZMC 25098	*Rhizobium* *radiobacter*	16S rDNA (OP964029)	Madaras (HU)	carrot	this study
SZMC 25100	*Rhodococcus* sp.	16S rDNA (OP964030)	Madaras (HU)	pepper	this study
SZMC 25101	*Rhodococcus* sp.	16S rDNA (OP964031)	Madaras (HU)	pepper	this study
SZMC 25102	*Arthrobacter oryzae*	16S rDNA (OP964032)	Madaras (HU)	pepper	this study
SZMC 25103	*Williamsia* *muralis*	16S rDNA (OP964033)	Madaras (HU)	pepper	this study
SZMC 25106	*Paenarthrobacter* sp.	16S rDNA (OP964034)	Čenej (SRB)	carrot	this study
SZMC 25107	*Streptomyces* sp.	16S rDNA (OP964035)	Čenej (SRB)	carrot	this study
SZMC 25108	*Variovorax* *paradoxus*	16S rDNA (OP964036)	Glozan (SRB)	carrot	this study
SZMC 25110	*Rhodococcus erythropolis*	16S rDNA (OP964037)	Temerin (SRB)	sweet potato	this study
SZMC 25116	*Priestia* sp.	16S rDNA (OP964038)	Čenej (SRB)	pepper	this study
SZMC 25117	*Pseudarthrobacter* sp.	16S rDNA (OP964039)	Čenej (SRB)	pepper	this study
SZMC 25124	*Rhodococcus* sp.	16S rDNA (OP964040)	Madaras (HU)	pepper	this study
SZMC 25125	*Olivibacter soli*	16S rDNA (OP964041)	Madaras (HU)	pepper	this study
SZMC 25126	*Priestia* *megaterium*	16S rDNA (OP964042)	Madaras (HU)	pepper	this study
SZMC 24978	*Bacillus* *mojavensis*	*gyrA* (OP970235)	Totovo Selo (SRB)	tomato	this study
SZMC 24981	*Bacillus* *velezensis*	*gyrA* (OK256098) *rpoB* (OK256116) fatty acid profiling	Totovo Selo (SRB)	pepper	[26]
SZMC 24982	*Bacillus* *velezensis*	*gyrA* (OK256099) *rpoB* (OK256117) fatty acid profiling	Totovo Selo (SRB)	pepper	[26]
SZMC 24983	*Bacillus* *velezensis*	*gyrA* (OK256100) *rpoB* (OK256118) fatty acid profiling	Totovo Selo (SRB)	pepper	[26]
SZMC 24984	*Bacillus* *velezensis*	*gyrA* (OK256101) *rpoB* (OK256119) fatty acid profiling	Čantavir (SRB)	pepper	[26]
SZMC 24985	*Bacillus* *velezensis*	*gyrA* (OK256102) *rpoB* (OK256120) fatty acid profiling	Čantavir (SRB)	pepper	[26]
SZMC 24986	*Bacillus* *velezensis*	*gyrA* (OK256103) *rpoB* (OK256121) fatty acid profiling	Čantavir (SRB)	tomato	[26]
SZMC 24995	*Bacillus* *velezensis*	*gyrA* (OK256104) *rpoB* (OK256122) fatty acid profiling	Čantavir (SRB)	tomato	[26]
SZMC 25001	*Bacillus* *mojavensis*	*gyrA* (OP970243)	Madaras (HU)	pepper	this study
SZMC 25003	*Bacillus* *velezensis*	*gyrA* (OP970244)	Madaras (HU)	pepper	this study
SZMC 25007	*Bacillus* *mojavensis*	*gyrA* (OP970245)	Čenej (SRB)	carrot	this study
SZMC 25018	*Bacillus* *mojavensis*	*gyrA* (OP970246)	Čenej (SRB)	tomato	this study
SZMC 25020	*Bacillus* *velezensis*	*gyrA* (OK256105) *rpoB* (OK256123)	Čenej (SRB)	tomato	[26]
SZMC 25856	*Ensifer* *adhaerens*	16S rDNA (MT950353) genome sequencing	Bačka region (SRB)	tomato	[27,28]
SZMC 25871	*Ensifer* *adhaerens*	16S rDNA (MT950357)	Bačka region (SRB)	tomato	[27]
SZMC 25863	*Pseudomonas* *resinovorans*	*rpoB* (MT955648)	Bačka region (SRB)	tomato	[27]
SZMC 25870	*Pseudomonas* *resinovorans*	*rpoB* (MT955649)	Bačka region (SRB)	tomato	[27]
SZMC 25872	*Pseudomonas* *resinovorans*	*rpoB* (MT955650) genome sequencing	Bačka region (SRB)	tomato	[27,28]
**Fungi**					
SZMC 25217	*Trichoderma* *ghanense*	*rpb2* (OP970255)	Čantavir (SRB)	tomato	this study
		*tef1α* (OP970248)			
SZMC 25220	*Trichoderma* *harzianum*	*rpb2* (OP970256)	Székkutas (HU)	carrot	this study
		*tef1α* (OP970249)			
SZMC 25228	*Trichoderma* *afroharzianum*	*rpb2* (OP970257)	Glozan (SRB)	carrot	this study
		*tef1α* (OP970250)			
SZMC 25231	*Trichoderma* *afroharzianum*	*rpb2* (OP970258)	Glozan (SRB)	carrot	this study
		*tef1α* (OP970251)			
SZMC 25235	*Trichoderma* *virens*	*rpb2* (OP970259)	Temerin (SRB)	batata	this study
		*tef1α* (OP970252)			
SZMC 25242	*Trichoderma* *guizhouense*	*rpb2* (OP970260)	Glozan (SRB)	tomato	this study
		*tef1α* (OP970253)			
SZMC 25242	*Trichoderma* *virens*	*rpb2* (OP970261) *tef1α* OP970254	Glozan (SRB)	tomato	this study

* Szeged Microbiology Collection; HU: Hungary, SRB: Serbia. GenBank accession numbers are shown in parentheses. “This study”: the identification of the respective strains and the submission of their sequences to GenBank was performed during this study.

**Table 2:** Minimum inhibitory concentration (MIC) values (µg mL^−1^) of the tested fungicides towards *Trichoderma* isolates.

	Carboxin	Bromoxinil	Thiram	Amitraz	Cyproconazole	Folpet	Imazalil	Penconazole
*T. afroharzianum*	250	250	125	7.8125	125	31.25	7.8125	31.25
SZMC 25228								
*T. afroharzianum*	250	250	125	7.8125	125	31.25	7.8125	31.25
SZMC 25231								
*T. ghanense*	250	125	250	62.5	62.5	250	62.5	125
SZMC 25217								
*T. guizhouense*	250	250	250	7.8125	125	250	15.625	125
SZMC 25242								
*T. harzianum*	250	250	62.5	250	31.25	62.5	3.9625	15.625
SZMC 25220								
*T. virens*	250	250	125	250	125	125	62.5	62.5
SZMC 25235								
*T. virens*	250	250	125	250	62.5	250	3.9625	7.8125
SZMC 26592								

**Table 3:** Growth of different bacterial isolates on nitrogen free medium.

Scheme 25080	SZMC * Numbers	Colony Size
*Arthrobacter globiformis*	SZMC 25080	+
*Arthrobacter globiformis*	SZMC 25081	+++
*Variovorax paradoxus*	SZMC 25084	++
*Nocardia* sp.	SZMC 25085	+
*Peribacillus simplex*	SZMC 25093	+
*Mycobacterium* sp.	SZMC 25094	++
*Agrobacterium tumefaciens*	SZMC 25098	++
*Rhodococcus* sp.	SZMC 25100	+
*Rhodococcus* sp.	SZMC 25101	+
*Arthrobacter oryzae*	SZMC 25102	++
*Williamsia limnetica*	SZMC 25103	+
*Paenarthrobacter* sp.	SZMC 25106	+
*Streptomyces* sp.	SZMC 25107	+
*Variovorax paradoxus NBRC 151*	SZMC 25108	++
*Rhodococcus erythropolis*	SZMC 25110	+
*Priestia* sp.	SZMC 25116	+
*Pseudarthrobacter* sp.	SZMC 25117	+
*Rhodococcus* sp.	SZMC 25124	+
*Olivibacter soli*	SZMC 25125	+
*Priestia megaterium*	SZMC 25126	+

* Szeged Microbiology Collection, +: poor growth, ++: moderate growth, +++: substantial growth.

**Table 4:** The highest concentration of pesticides tolerated by *Pseudomonas resinovorans* SZMC 25872.

Pesticide	Highest Tolerated Concentration, µg mL^−1^
*Herbicides*	
Bensulfuron-methyl	>25
Chlorotoluron	>25
Chlorpropham	>25
Chlorsulfuron	>25
Cinosulfuron	>25
Diuron	>25
Dimethachlor	>25
Fenuron	>25
Ethoxysulfuron	12.5
Glyphosate	>25
Glyphosate (*Glialka Star*)	>25
Isoproturon	>25
Linuron	>25
MCPA	>25
Primisulfuron-methyl	>25
Propham	>25
Triasulfuron	>25
2,4-D	>25
*Fungicides*	
Captan	6.25
Carbendazim	>25
Carboxin	>25
Fenarimol	>25
Flutriafol	>25
Imazalil	>25
Mancozeb	<6.25
Maneb	<6.25
Penconazole	>25
Tebuconazole	>25
Thiabendazole	>25
Thiram	6.25
Thiophanate-methyl	>25
Zineb	6.25
*Insecticide*	
Diflubenzuron	>25

## Data Availability

The data of this study are available from the corresponding author.
